# Elevation of IL-6 in the allergic asthmatic airway is independent of inflammation but associates with loss of central airway function

**DOI:** 10.1186/1465-9921-11-28

**Published:** 2010-03-08

**Authors:** Wendy A Neveu, Jenna L Allard, Danielle M Raymond, Lorraine M Bourassa, Stephanie M Burns, Janice Y Bunn, Charles G Irvin, David A Kaminsky, Mercedes Rincon

**Affiliations:** 1 Department of Medicine, Division of Immunobiology, University of Vermont, Burlington, 05405, USA; 2 Pulmonary and Critical Care Medicine, University of Vermont, Burlington, 05405, USA; 3 Department of Medical Biostatistics, University of Vermont, Burlington, 05405, USA

## Abstract

**Background:**

Asthma is a chronic inflammatory disease of the airway that is characterized by a Th2-type of immune response with increasing evidence for involvement of Th17 cells. The role of IL-6 in promoting effector T cell subsets suggest that IL-6 may play a functional role in asthma. Classically IL-6 has been viewed as an inflammatory marker, along with TNFα and IL-1β, rather than as regulatory cytokine.

**Objective:**

To investigate the potential relationship between IL-6 and other proinflammatory cytokines, Th2/Th17 cytokines and lung function in allergic asthma, and thus evaluate the potential role of IL-6 in this disease.

**Methods:**

Cytokine levels in induced sputum and lung function were measured in 16 healthy control and 18 mild-moderate allergic asthmatic subjects.

**Results:**

The levels of the proinflammatory biomarkers TNFα and IL-1β were not different between the control and asthmatic group. In contrast, IL-6 levels were specifically elevated in asthmatic subjects compared with healthy controls (p < 0.01). Hierarchical regression analysis in the total study cohort indicates that the relationship between asthma and lung function could be mediated by IL-6. Among Th2 cytokines only IL-13 (p < 0.05) was also elevated in the asthmatic group, and positively correlated with IL-6 levels (r_S _= 0.53, p < 0.05).

**Conclusions:**

In mild-moderate asthma, IL-6 dissociates from other proinflammatory biomarkers, but correlates with IL-13 levels. Furthermore, IL-6 may contribute to impaired lung function in allergic asthma.

## Background

Asthma is a chronic inflammatory disease with pathological changes that occur in the lung such as airway eosinophilia, mucus metaplasia and mucus hypersecretion. These changes are associated with the development of a CD4^+ ^Th2 type of immune response in the lung. This immune response is characterized by the secretion of IL-4, IL-5 and IL-13, with minimal production of the Th1 type of cytokines (e.g. IFNγ) [1]. More recent studies have also shown an association of CD4^+ ^Th17 type of immune response with allergic airway inflammation, but the contribution of Th17 cells and IL-17 to asthma pathology is unclear [2-4]. Since the cytokine environment is one important factor that influences the fate of effector CD4^+ ^T cells, it is possible that cytokines produced by structural elements in the lung influence the local immune response. Although not part of the immune system, lung epithelial cells can also contribute to the type of immune response by secreting specific cytokines. One of the cytokines that is produced by lung epithelial cells is IL-6 [5,6] and increased production of IL-6 by lung epithelial cells has been found in asthmatic patients relative to control subjects [7,8].

IL-6 is a pleotropic cytokine that, together with TNFα and IL-1β, has been traditionally considered as a biomarker of ongoing inflammation more than as a regulatory cytokine with potential to modulate the immune response [9]. However, recent studies suggest that IL-6 plays an important role in determining the type of adaptive immune response, primarily in the differentiation of effector CD4^+ ^T cells [10]. Specifically, IL-6 has been shown to promote Th2 differentiation of CD4^+ ^T cells while suppressing Th1 differentiation through independent pathways [10]. IL-6 can also modulate the intensity of the immune response by inhibiting T regulatory (Treg) cell development [11]. More recently, a number of studies have shown that IL-6, together with TGF-β promotes the generation of murine Th17 cells [12-14]. In humans however, the role of IL-6 in Th17 differentiation is somewhat controversial since some studies suggest that IL-6 is not required for Th17 development [15,16] while other studies suggest that IL-6 synergizes with IL-1β to promote Th17 differentiation [17]. Thus, IL-6 may be a key factor in determining the balance of CD4^+ ^T cells in becoming Treg or inflammatory Th17 cells.

Taken together, these findings suggest that rather than being a marker of ongoing inflammation, IL-6 may have a more functional role. To date, the animal studies addressing the role of IL-6 in allergic airway inflammation have provided conflictive results. Studies using IL-6 deficient mice suggest IL-6 protects against airway inflammation, while studies using neutralizing antibodies suggest that IL-6 promotes allergic airway inflammation [18,19]. Moreover, there has been relatively less interest in the pathobiology of IL-6 in human asthma. In order to investigate whether IL-6 might be more than an inflammatory marker in asthma, we determined the levels of IL-6, TNFα and IL-1β in induced sputum and then related this to lung function in a group of mild-moderate allergic asthmatic and healthy control subjects.

## Methods

### Subjects

We recruited mild-moderate allergic asthmatic and healthy control adult subjects. Individuals were defined as mild-moderate asthmatics according to the National Institutes of Health Expert Panel Report 2 guidelines [20]. The protocol was approved by the University of Vermont Institutional Review Board and informed consent was obtained. Asthmatic subjects (n = 18) had no history of other cardiopulmonary diseases, were nonsmokers for at least 3 years and had less than a 5 pack year history, FEV_1 _>70% predicted, exhibited positive methacholine induced airway hyperresponsiveness defined as a provocation concentration of methacholine causing a 20% fall in FEV_1 _(PC20<8 mg/ml), and were atopic by positive skin prick testing to one or more of 6 common Northeast regional allergen extracts. The wheal flare response at sites of allergen exposure were compared to negative control (glycerated saline) and positive control (10 mg/ml histamine) skin prick sites in order to determine atopic status. Healthy controls (n = 16) were negative for allergies and respiratory diseases, exhibited normal pulmonary function tests and PC20>16 mg/ml. A subgroup of asthmatic subjects were taking inhaled corticosteroids daily in combination with inhaled β-agonists (n = 9). Two out of the 9 asthmatic patients using this treatment were also on antileukotrienes. The remaining subjects within the total asthmatic group took inhaled β-agonists as needed (n = 6) or were on no treatment at all (n = 3).

### Study Design

Subjects underwent lung function testing and methacholine challenge as previously described [21,22]. At the first visit, we obtained history and performed lung spirometry and methacholine challenge. Skin prick testing to *Dermatophagoides pteronyssinus *and *D. farinae*, cat dander, grass and tree pollen, and *Alternaria alternata *was conducted to establish atopic status at visit 2. Lung spirometry and induced sputum collection was performed at visit 3. Asthmatic subjects using albuterol and salmeterol were instructed to discontinue drug use 8 h and 24 h, respectively, prior to lung function testing.

### Sputum Induction and Cytokine Analysis

Sputum samples were collected and processed as previously described [23]. Briefly, subjects were treated with a bronchodilator and then administered increasing concentrations of nebulized hypertonic saline solution. At select intervals during the procedure subjects were encouraged to expectorate airway secretions through a deep cough maneuver. Sputum plugs collected during induction were treated with a volume of a 1:10 dilution of dithiotreitol (DTT) (Sputolysin; Calbiochem) in PBS that was four times their weight. Samples were centrifugated at 400 g for 10 min. Supernatants were then collected and stored at -80°C until cytokine analysis. Cytospins were prepared from the remaining cell pellets collected from the sputum preparation and analyzed by the Giemsa staining method. Those samples that demonstrated greater than 20% squamous cell contamination were excluded. The majority of our sputum preparations from healthy and asthmatic patients contained <8% squamous cells. Analysis of lung epithelial cells showed similar frequency of these cells in healthy (4.3% +/- 5.2 SD) and asthmatic (4.8% +/- 5.7 SD) groups.

Sputum supernatants were analyzed for multiple cytokines in triplicate using the Human 17-Plex Panel (Bio-Rad). The Bio-plex system was chosen for cytokine analysis since it has been validated by other studies for cytokine detection in sputum supernatant [23,24]. Cytokine standards were reconstituted in a PBS-DTT dilution buffer as a way to normalize the sputum samples since a previous report has shown that DTT may interfere with the detection of select cytokines by ELISA [25], although we have not found that to be the case for the cytokines that we measured. The cytokine mix provided by the manufacturer was serially diluted for construction of the standard curve. PBS-DTT dilution buffer without cytokines was used as negative control. The collected data was analyzed using the Bio-Plex Manager Software (Bio-Rad). The limit of detection for the Bio-plex assay ranged from 0.2-19.3 pg/ml according to the manufacturer.

### Statistical Analysis

Cell counts were expressed as median (interquartile range, IQR). Due to the skewed nature of the distributions, non-parametric techniques were used to analyze all data. Differences between asthmatic patients and controls were examined using the Wilcoxon ranked-sum test, while Spearman's rank correlation coefficients (r_S_) were computed for correlations. Particular interest focused on whether cytokine levels could serve as mediators in the relationship between disease status (e.g. asthma) and lung function. Specifically, variables are said to act as mediators when they represent a mechanism through which the independent variable of interest is able to influence the dependent variable of interest [26]. This model assumes that there are two effects of the independent variable: the direct effect and an indirect effect acting through the mediator. In order for a variable to function as a mediator, it must 1) be significantly related to the independent variable, 2) be significantly related to the dependent variable, and 3) when both the independent variable and the potential mediator are included in the same model, a previously significant relationship between the independent and dependent variables is reduced or eliminated. In order to explore the potential mediation effects of the various cytokines, we performed a hierarchical regression analysis, beginning with a series of simple regression analyses conducted to examine the relationship between disease status and cytokine levels, cytokine levels and lung function and disease status and lung function. In the event that the simple regression analyses suggest statistically significant relationships, a multiple regression analysis is performed with lung function as the dependant variable and both disease status and cytokine levels as independent variables. All continuous variables were transformed to ranks prior to analysis [26]. A p < 0.05 was considered significant.

## Results

Subject demographics and lung function data for the healthy control and mild-moderate asthmatic groups are reported in Table 1. The mean age and male to female ratio was similar in both groups. At baseline, the asthmatic group exhibited a decrease in FEV_1 _in comparison to control subjects. All of the asthmatic subjects were atopic to at least one of the allergens tested in this study, with *Dermatophagoides *being the most prevalent. Fifty-percent of the group was sensitized to multiple allergens.

**Table 1:** Patient Demographics

Criteria	Control Subjects	Mild to Moderate Asthmatic Subjects
Number	16	18

Sex (M/F)	6/10	7/11

Age, yr	25.2 ± 6.0	25.9 ± 9.4

Antileukotrienes	0	2

Inhaled Steriods	0	9

FEV_1 _% predicted	105.6 ± 11.4	90.2 ± 14.3^‡^

FEV_1_/FVC % predicted	101.5 ± 4.1	87.6 ± 9.8^§^

PC20 (mg/ml)	>16	1.0 (0.48-2.1)^†^

Squamous cells (%)	1.5 (3.8)*	2.0 (6.0)*

Lung Epithelial cells (%)	2.0 (5.3)*	2.5 (3.0)*

Eosinophils (%)	1.1 (3.0)*	2.0 (6.0)*

Neutrophils (%)	19.85 (21.1)*	6.95 (17.8)*

Values are reported as means ± SD unless otherwise indicated. Definition of abbreviations: F = females, FEV_1 _= forced expiratory volume in 1 second, FEV_1_/FVC = FEV_1_/forced vital capacity, M = males, PC20 = provocation concentration of methacholine causing a 20% fall in FEV_1_. The percentage of eosinophils and neutrophils are reported for induced sputum.

^†^Geometric mean (range)

*Median (interquartile range)

^‡^p = 0.004, asthmatic vs. control group

^§^p < 0.0001, asthmatic vs. control group

**Figure 1:**
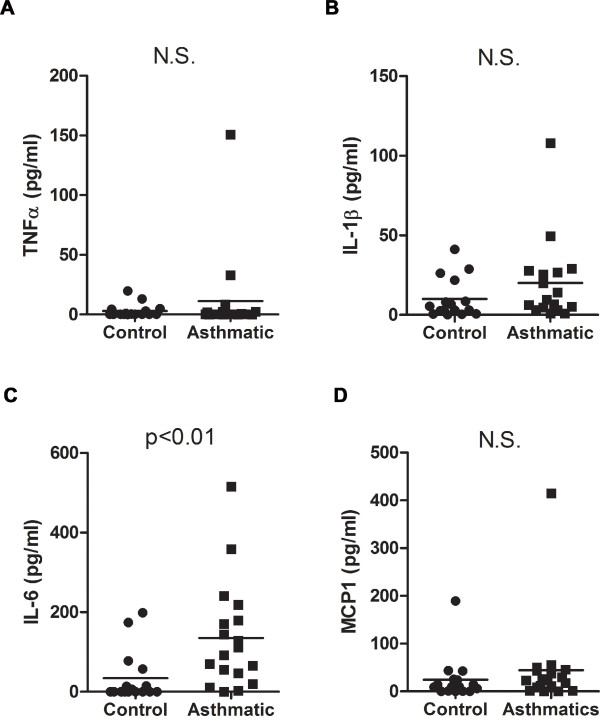
**IL-6 but not TNFα or IL-1β is elevated in induced sputum of mild-moderate asthmatic subjects**. (A) TNFα, (B) IL-1β, (C) IL-6 and (D) MCP-1 levels were analyzed in induced sputum of mild-moderate asthmatic subjects (n = 18) and healthy controls (n = 16).

Examination of cytokine levels in induced sputum from the study groups showed that TNFα (Fig. 1A) and IL-1β (Fig. 1B) levels were relatively low in the asthmatics and were not statistically different from the levels in the control group. No correlation between the levels of these cytokines and medical treatment was found in the asthmatic group (data not shown). Interestingly, in contrast to TNFα and IL-1β, IL-6 levels were significantly higher in the asthmatic group relative to control subjects (Fig. 1C). Within the asthmatic population no difference in IL-6 levels could be detected when asthmatics were separated into groups based on medical treatment (e.g. β-agonists alone or inhaled corticosteroids in combination with β-agonists) and those not on medication (see Additional File 1). In addition to TNFα and IL-1β, there was no significant difference in levels of the proinflammatory cytokine monocyte chemoattractant protein-1 (MCP-1) [27] in the induced sputum between asthmatics and controls (Fig. 1D).

**Figure 2:**
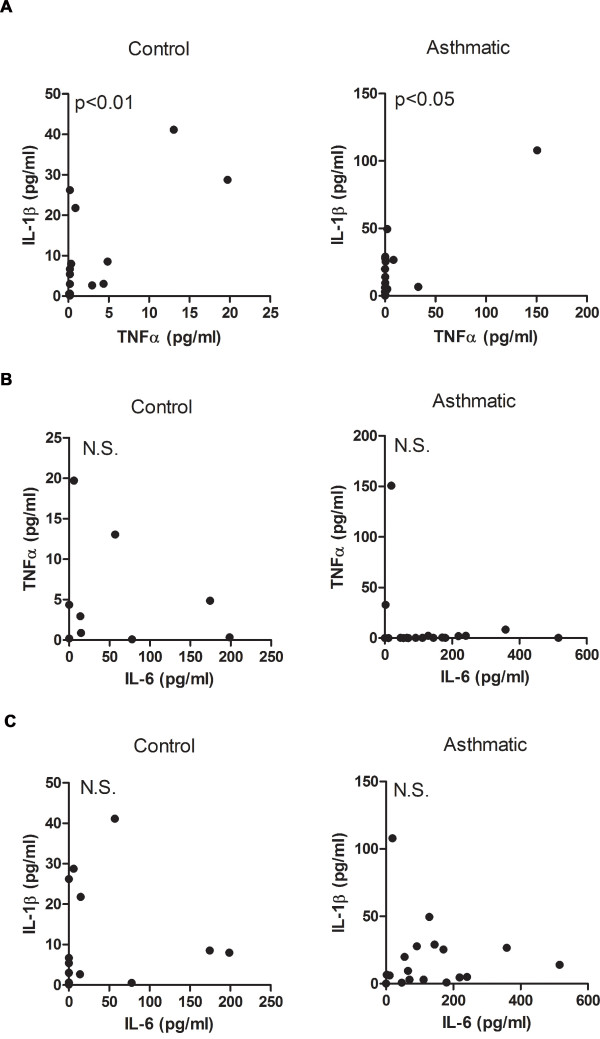
**While TNFα correlates with IL-1β in induced sputum, neither correlates with IL-6 in mild-moderate asthmatic subjects**. (A) Correlative analysis of TNFα and IL-1β levels in control (r_s _= 0.64, p < 0.01) and asthmatic subjects (r_s _= 0.49, p < 0.05). (B) Correlative analysis of IL-6 and TNFα levels in control (r_s _= 0.46, p = 0.08) and asthmatic subjects (r_s _= 0.29, p = 0.24). (C) Correlative analysis of IL-6 and IL-1β levels in control (r_s _= 0.38, p = 0.15) and asthmatic subjects (r_s _= 0.18, p = 0.48).

Since IL-6 has been shown to be involved in the inflammatory response, we analyzed its association with TNFα and IL-1β. Comparative analysis of IL-6, TNFα and IL-1β showed a positive correlation between TNFα and IL-1β in both control and asthmatic groups (Fig. 2A), but there was no correlation between IL-6 and TNFα (Fig. 2B) or IL-6 and IL-1β (Fig. 2C).

**Figure 3:**
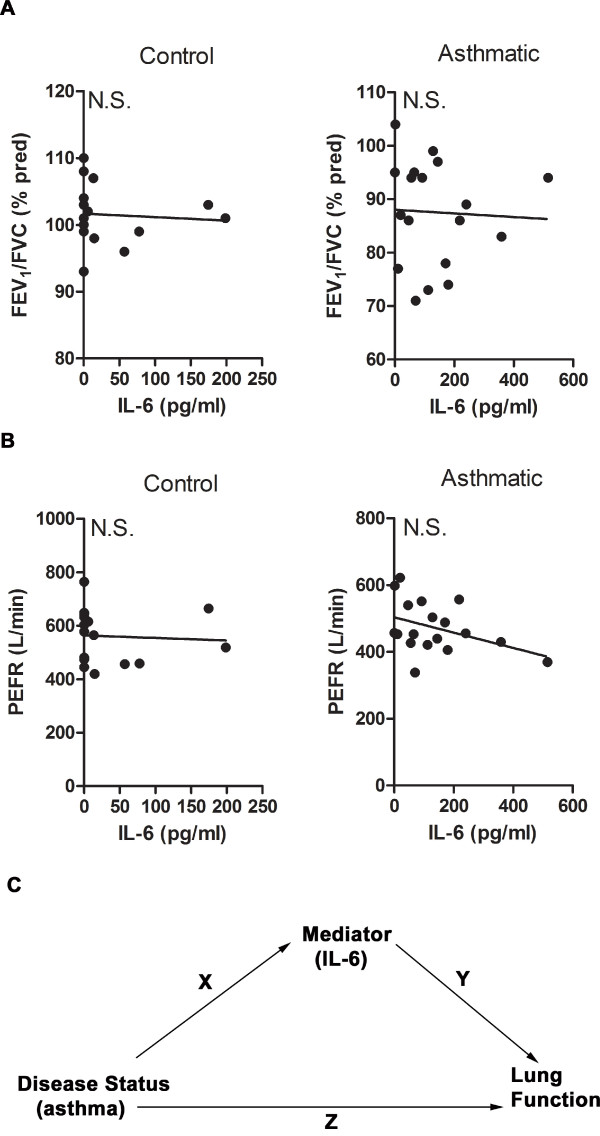
**Relationship of IL-6 with lung function in mild-moderate asthmatic subjects**. (A) Correlative analysis of sputum IL-6 levels and FEV_1_/FVC ratio in control (r_s _= -0.18, p = 0.50) and asthmatic (r_s _= -0.23, p = 0.37) subjects. (B) Correlative analysis of sputum IL-6 levels with PEFR in control (r_s _= -0.26, p = 0.34) and asthmatic (r_s _= -0.37, p = 0.13) subjects. (C) Schematic representation of the hierarchical regression analysis in the total cohort. Path X is the correlation between IL-6 levels and disease status (asthma). Path Y is the correlation between IL-6 levels and lung function. Path Z is the correlation between disease status and lung function.

We also examined the potential link of lung function with IL-6 levels in induced sputum. Pulmonary function testing was performed on the same day that the induced sputum was collected. Since most of the patients in this study have mild asthma, with only a few of them exhibiting signs of moderate disease, the FEV_1_% predicted in this group was clustered within a narrow range (Table 1). No statistically significant correlation could be found between FEV_1_% predicted (data not shown), or FEV_1_/forced vital capacity (FVC)% predicted with IL-6 levels within the control or asthmatic groups (Fig. 3A). Although it did not reach significance, there was a trend towards an inverse correlation between the peak expiratory flow rate (PEFR) and IL-6 levels in the asthmatic group relative to the control group (Fig. 3B).

Considering that the number of subjects per group was limited, to further examine a potential link between IL-6 and impaired lung function we used the hierarchical regression analysis in the total cohort (combining control and asthmatic subjects). This type of analysis builds from simple to multiple regression analyses to investigate the potential contribution of mediators (e.g. IL-6) to an already established relationship (e.g. asthma and impaired lung function) (Fig. 3C). As expected from the FEV_1_/FVC% predicted and PEFR values in asthmatic and control subjects (Fig 3A and 3B), simple regression analysis in the total cohort showed a significant inverse correlation between disease status (asthma) and lung function (Fig. 3C and Table 2, Path Z). Simple regression analysis also showed a direct correlation between IL-6 levels and disease status (Table 2, Path X), as predicted from the results in Fig. 1C. Moreover, simple regression analysis of IL-6 and lung function (Fig. 3C, Path Y) showed a statistically significant inverse relationship between IL-6 and lung function, with increased levels of IL-6 related to decreased FEV_1_/FVC% predicted and PEFR values (Table 2, Path Y). We then performed multiple regression analyses to explore whether IL-6 could be a mediator of the impaired lung function in asthma (Figure 3C, Paths X and Y). If the relationship between disease status and lung function is mediated by IL-6, a decrease in the strength (β-coefficient) of the relationship between disease status and lung function or a complete loss of the statistical significance in this relationship is expected in the multiple regression analysis [24]. The value of the regression parameter describing the relationship between FEV_1_/FVC% predicted and disease status was decreased in the multiple regression analysis (β = -11.69) compared with the value in the simple regression analysis (β = -13.58), but it remained statistically significant (p = 0.0005) (Table 2). In contrast, the statistically significant relationship between PEFR and disease status in the simple regression analysis (p = 0.0052) disappeared when IL-6 was included in the multiple regression model (p = 0.09) (Table 2). These results suggest that the relationship between disease status and lung function could be mediated by IL-6, particularly when lung function is measured as PEFR.

**Table 2:** Analysis of IL-6 in the relationship between disease status and lung function

Simple Regression Analyses		
**Path X**		
Disease status (asthma)	IL-6 (mediator) 10.68^a ^(2.86)^b^ *p < 0.0007*	

**Path Y**		
	Lung function Parameters	
	
IL-6 (mediator)	FEV1/FVC% predicted	PEFR
		
	-0.5 (0.16) *p = 0.0025*	-0.45 (0.16) *p = 0.0088*

**Path Z**		
	Lung function Parameters	
	
Disease status (asthma)	FEV1/FVC% predicted	PEFR
		
	-13.58 (2.51) *p < 0.0001*	-9.21 (3.07) *p = 0.0052*

**Multiple Regression Analyses**		

	Lung function Parameters	

	FEV1/FVC% predicted	PEFR
		
Disease status (asthma)	-11.69 (2.99) *p = 0.0005*	-6.34 (3.62) *p = 0.09*
IL-6 (mediator)	-0.177 (0.15) *p = 0.2594*	-0.27 (0.19) *p = 0.1599*

a: β-coefficient

b: standard errors

**Figure 4:**
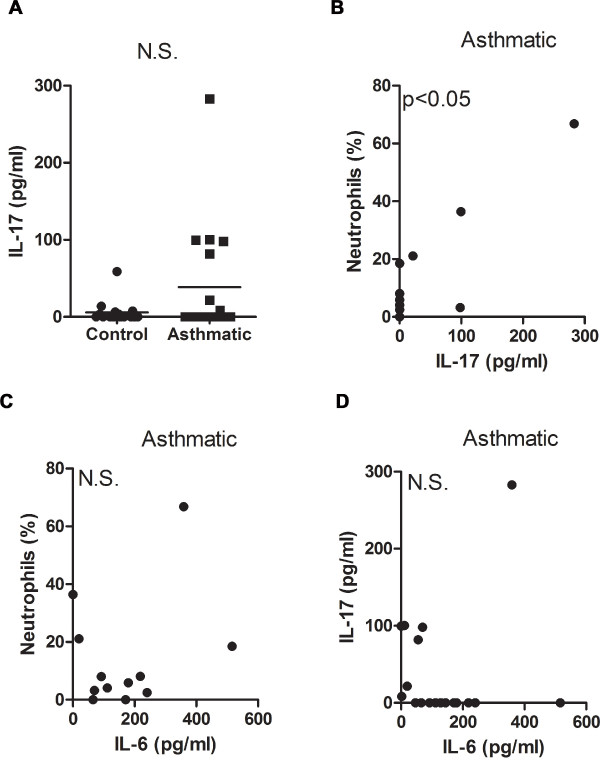
**IL-17 levels in mild-moderate asthmatic subjects and its relationship to IL-6**. (A) IL-17 levels were analyzed in control and asthmatic subjects. (B) Correlative analysis of IL-17 levels with percentage of neutrophils in asthmatic subjects (r_s _= 0.60, p < 0.05). (C) Correlative analysis of IL-6 levels with percentage of neutrophils in asthmatic subjects (r_s _= 0.07, p = 0.83). (D) Correlative analysis of IL-6 and IL-17 levels in asthmatic subjects (r_s _= -0.45, p = 0.06).

The dissociation of IL-6 from other proinflammatory markers (e.g. TNFα and IL-1β) and the well-established role of IL-6 in the differentiation of CD4^+ ^T cells, led us to examine the presence of specific cytokines for defined effector T cell subsets. Recent studies point to a potential role of Th17 cells in airway inflammation in asthma. IL-6 has been shown to promote Th17 cell differentiation in mouse models [12-14], although its contribution to human Th17 cell development remains controversial [15-17]. We therefore examined IL-17 (IL-17A) levels in induced sputum from control and mild-moderate asthmatic subjects. Although there was no statistically significant difference in IL-17 levels between the control and asthmatic groups (Fig. 4A), there seemed to be a subset of asthmatic patients that had increased IL-17 levels. Since IL-17 has been associated with neutrophil recruitment we examined the association of this cytokine with neutrophils in induced sputum. IL-17 levels in the asthmatic group showed a strong positive correlation with airway neutrophilia (Fig. 4B). However, IL-6 levels in the asthmatic patients did not correlate with the presence of neutrophils in the airway, further indicating the dissociation of IL-6 with inflammation (Fig. 4C). Furthermore, IL-6 levels within the asthmatic population did not correlate with IL-17 levels (Fig. 4D). Thus, in mild-moderate allergic asthma there is no clear association between IL-6 and the IL-17/neutrophils axis.

**Figure 5:**
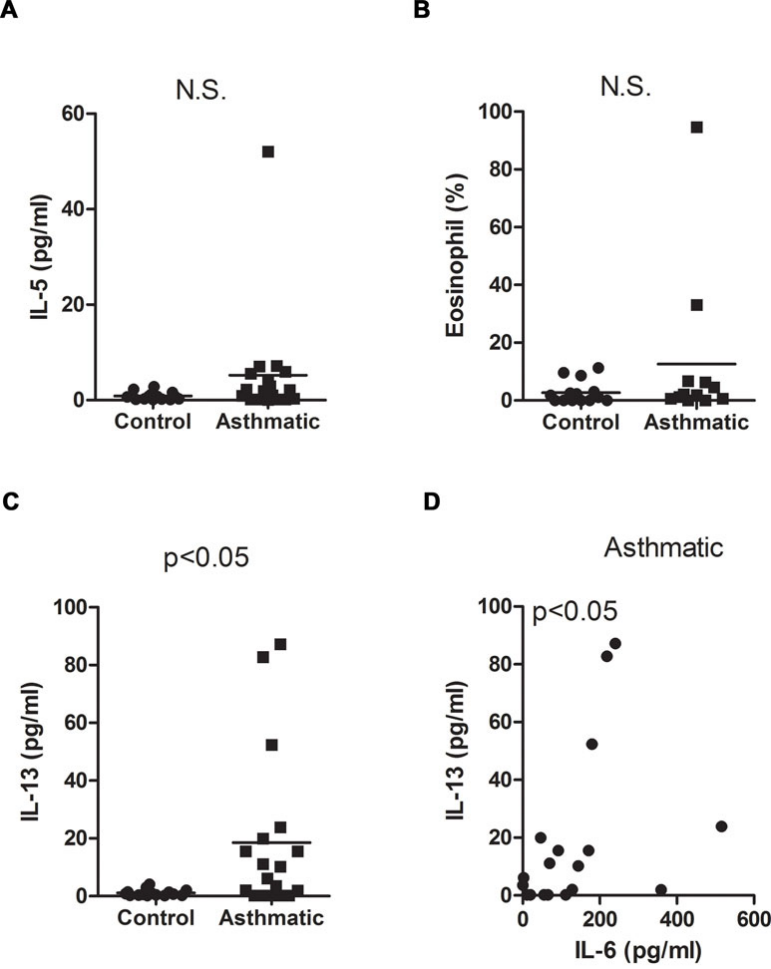
**Selective elevation of IL-13 in mild-moderate asthmatic subjects compared with healthy control subjects**. (A) IL-5 levels and (B) percentage of eosinophils in induced sputum were analyzed in the control and asthmatic groups. (C) IL-13 levels were analyzed in control and asthmatic subjects. (D) Correlative analysis of IL-6 and IL-13 levels in the asthmatic group (r_s _= 0.53, p < 0.05).

IL-6 is also known to promote Th2 polarization while suppressing Th1 differentiation [10]. We therefore examined the presence of Th2 (IL-4, IL-5, IL-13) and Th1 (IFNγ) cytokines in induced sputum. IFNγ and IL-4 levels were negligible in most of the subjects from both groups and, therefore, they could not undergo statistical analysis (data not shown). Low levels of IL-5 could be detected, but there was no significant difference between the control and asthmatic groups (Fig 5A). The lack of increased IL-5 levels in the mild-moderate asthmatic subjects correlated with the lack of a strong eosinophilic response in the airway of the asthmatic patients since the levels of eosinophils in this group were similar to those in the control subjects (Fig. 5B). In contrast to IL-5, IL-13 levels were clearly increased in asthmatic subjects compared with controls (Fig. 5C). Thus, both IL-6 and IL-13 are selectively elevated in the airway of mild-moderate asthmatic patients compared with control subjects. Furthermore, comparative analysis of these cytokines shows IL-13 levels positively correlate with elevated IL-6 levels in the asthmatic population (Fig. 5D). These results further support our recent study showing IL-6 is required for IL-13 production in an experimental mouse model of allergic asthma [28]. However, unlike IL-6, analysis of IL-13 in the total cohort showed no correlation of IL-13 with FEV_1_/FVC% predicted (r_s _= -0.20, p = 0.26) or PEFR (r_s _= -0.05, p = 0.76).

## Discussion

Over the last decade increasing evidence has shown that IL-6 can modulate the adaptive immune response during early T cell activation. Its ability to influence various CD4^+ ^effector T cell subsets suggests that IL-6 may play a functional role in diseases that are mediated by the activity of these cells. Despite this body of knowledge, IL-6 is still considered as a non-specific inflammatory marker with other cytokines such as IL-1β and TNFα in a variety of immune-mediated diseases. IL-6 is viewed as a product of an ongoing innate immune response, primarily as a factor derived by activated macrophages and/or dendritic cells. However, IL-6 can be produced by cells other than hematopoietic cells, such as epithelial cells, endothelial cells and fibroblasts, in response to specific stimuli [9]. Thus, IL-6 could be produced in the absence of inflammatory cells, and might be a sign of tissue specific pathology. A good example of this is Rheumatoid Arthritis where IL-6 has long been used as a biomarker of disease severity. However, recent clinical trials with tocilizumab have shown that IL-6 contributes to both disease severity and progression [29,30]. This opens up the possibility that in other diseases in which IL-6 is also elevated, this cytokine may not only be an indicator of inflammation, but contributes to the initiation and/or progression of disease.

IL-6 has been shown to be increased in chronic inflammatory diseases of the airway. Increased IL-6 levels in induced sputum have been found in patients with severe-very severe Chronic Obstructive Pulmonary Disease (COPD) relative to mild-moderate COPD patients [31]. In asthma, several reports have also shown elevated IL-6 levels in BALF and serum as well as increased IL-6 secretion from lung epithelial cells collected from asthmatics [7,8,32,33]. In this study we report that IL-6 levels in induced sputum from mild-moderate allergic asthmatic patients are significantly increased compared with levels in control subjects and that neither TNFα, IL-1β or MCP-1 was increased in the asthmatic population. Furthermore, we show that there is a clear dissociation of IL-6 levels with both IL-1β and TNFα in the asthmatic group. On the other hand, IL-1β and TNFα associate with each other. These results, together with a lack of significant eosinophilia and neutrophilia in asthmatic subjects, suggest that there is relatively no inflammation, as classically defined, within this group of asthmatic patients and that IL-6 detected in the lung microenvironment is not a result of an inflammatory response. Instead, IL-6 may be indicative of an ongoing process that contributes to the underlying disease in these patients.

Although no IL-4 could be detected in the induced sputum of asthmatic patients and IL-5 levels were not different between the asthmatic and control groups, the other classical Th2 cytokine IL-13 was selectively increased in the asthmatic group. We have previously reported that IL-6 promotes the differentiation of mouse CD4^+ ^T cells into Th2 cells that produce IL-4, but it does not induce IL-5 production [34]. We have recently shown that a mouse model of allergic airway inflammation induced by *Aspergillus fumigatus *extracts lung exposure induces rapid production of IL-6 in the airway and that IL-6 is critical for IL-13 production in the lung microenvironment [28]. The correlation of IL-6 and IL-13 levels in asthmatic patients further support the interaction between these two cytokines. One of the major functions of IL-13 is to promote the production of mucus by airway epithelial cells [35-37]. Mucus hypersecretion can also contribute to asthma pathology. Interestingly, minimal mucus production could be detected in IL-6 deficient mice exposed to *A. fumigatus *relative to wild type mice, correlating with their impaired IL-13 production [28]. Studies in mouse models have also suggested that IL-13 can modulate airway hyperresponsiveness by increasing the response of airway smooth muscle cells to specific bronchoconstrictors [36-38]. Thus, the selective elevation of IL-13 relative to other Th-derived cytokines in this group of asthmatic patients could contribute to pathological changes in the airway such as mucus hypersecretion and smooth muscle remodeling that leads to structural changes in the large conducting airway.

While the role of the Th17 immune response in allergic airway inflammation is still under investigation, early studies in humans have shown that expression of IL-17 within the airways is associated with increased influx of neutrophils to the lung and asthma severity [4]. Since the majority of asthmatic subjects in our study have relatively mild disease, it was not surprising that IL-17 levels within this group were not statistically different from the controls. Nevertheless, we show a direct correlation between sputum IL-17 levels and the presence of neutrophils in the airway was observed in accordance with previous reports [39]. Although IL-17-producing mononuclear cells have been found in the lungs of severe asthmatic patients [4], their identification as bona fide Th17 CD4^+ ^T cells has not been fully demonstrated. While mouse studies have shown IL-6 to be an important factor in the differentiation of Th17 cells its role in the development of the Th17 lineage in humans is less clear. Some studies have shown that human peripheral CD4^+ ^T cells fail to differentiate in the presence of IL-6 and TFGβ [15,16] while other reports show that IL-6 augments IL-1β-induced IL-17 production from human CD4^+ ^T cells [17]. Our studies suggest that there is not a clear association of IL-6 with IL-17 in the airway of mild-moderate allergic asthmatics.

The selective presence of IL-6 in the sputum of asthmatic patients without active inflammation suggests a potential role of this cytokine in the maintenance of the disease instead of merely being a non-specific marker of inflammation. In support of this concept, recent studies have shown that sputum IL-6 levels inversely correlates with lung function as determined by FEV_1_% predicted and FEV_1_/FVC in patients with COPD [40]. Previous studies in obese asthmatics that include a substantial number of subjects with severe asthma have shown an inverse correlation of IL-6 in induced sputum with FEV_1_% predicted in a cohort of asthmatics [23,41]. In the current study, we show that IL-6 does not correlate with FEV_1_/FVC% predicted, but there is a trend towards an inverse correlation with PEFR in the asthmatic group. Furthermore, the hierarchical regression analysis in the total cohort shows that the relationship between disease status and lung function (as determined preferentially by PEFR) is mediated by IL-6. Thus, IL-6 can contribute to increase airway obstruction in asthma. This type of statistical analysis requires a significant relationship between the mediator (cytokines) and disease status, and between the mediator and lung function. Because the other examined cytokines were not statistically significantly related to disease status (e.g. IL-1β, TNFα, MCP-1, IL-5, and IL-17) and/or lung function (e.g. IL-13) in the total study cohort, the final step in the analysis exploring a mediator function (i.e. the multiple regression analysis) was not performed.

In addition to modulating the type of CD4^+ ^T cell response, the current data suggest that IL-6 could also influence lung physiology by promoting an increase in airway wall thickness, subepithelial fibrosis, and smooth muscle hypertrophy and proliferation, as supported by animal and human studies [42-45]. Accumulation of IL-6 within the mild-moderate asthmatic group may reflect cellular processes that are occurring within the larger airways since previous studies have shown that induced sputum collection occurs within the central rather than peripheral airways [46]. The inverse association that IL-6 has with measures of central airway function such as PEFR and, to a lesser extent with FEV_1_/FVC, in the total study cohort suggests central airway remodeling is mediated by IL-6. Undoubtedly, the significance of elevated sputum IL-6 in allergic asthma remains unclear. Future studies on the role of IL-6 in the pathophysiology of asthma are warranted and depending on the results from these studies, IL-6 could be a potential therapeutic target in this disease.

## Conclusions

In summary, this study has shown that IL-6, rather than TNFα or IL-1β, is elevated in induced sputum from mild-moderate allergic asthmatic subjects, and that IL-6 positively correlates with increased IL-13 levels within this patient population. Furthermore, our data also suggests that IL-6 contributes to impaired lung function in allergic asthma. IL-6 is therefore more than a proinflammatory marker in the lung and may play a role in the pathophysiology of asthma.

## List of Abbreviations

(FEV_1_): Forced expiratory volume in 1 second; (FVC): Forced vital capacity; (PEFR): Peak expiratory flow rate; (PC20): Provocation concentration of methacholine causing a 20% fall in FEV_1_; (MCP-1): Monocyte chemoattractant protein-1.

## Competing interests

The authors declare that they have no competing interests.

## Authors' contributions

WN carried out the sputum cell counts, participated in sputum cytokine analysis and data analysis, and wrote this manuscript. JA participated in the sputum processing and sputum cytokine analysis. DR participated in sputum processing and cytology. LB, SB, and DK carried out patient recruitment, skin prick testing, pulmonary function testing, and sputum induction. MR, DK and CI participated in the design of the study and data analysis. JB conducted the statistical analysis for this study. All authors have read and approved of the final manuscript.

## Supplementary Material

Additional file 1 **Distribution of IL-6 levels in induced sputum by asthma treatment**. This figure represents IL-6 levels in induced sputum of mild-moderate asthmatic subjects on no medication (black circle), inhaled β-agonists as needed (black square), or inhaled corticosteroids (ICS) in combination with β-agonists (black triangle). Click here for file
