# Supplementary figures and images for: Elevation of IL-6 in the allergic asthmatic airway is independent of inflammation but associates with loss of central airway function
Source: Respir Res. 2010 Mar 8;11(1):28. doi: 10.1186/1465-9921-11-28 (PMC2842243; doi:10.1186/1465-9921-11-28)

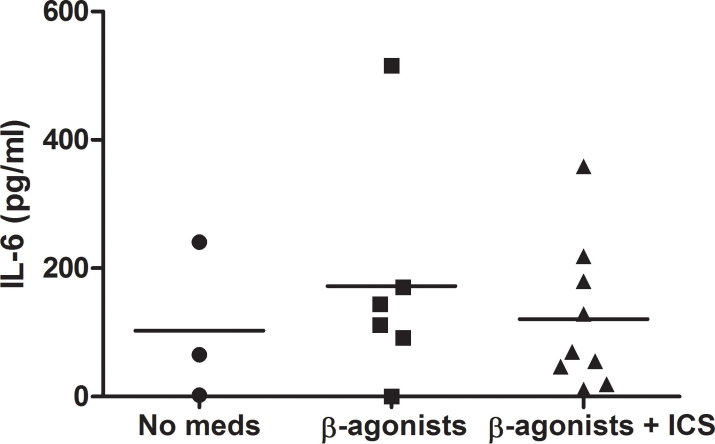

Supplement: Additional file 1 — Distribution of IL-6 levels in induced sputum by asthma treatment. This figure represents IL-6 levels in induced sputum of mild-moderate asthmatic subjects on no medication (black circle), inhaled β-agonists as needed (black square), or inhaled corticosteroids (ICS) in combination with β-agonists (black triangle). [file 1465-9921-11-28-S1.PDF]
